# An Appraisal of Incremental Learning Methods

**DOI:** 10.3390/e22111190

**Published:** 2020-10-22

**Authors:** Yong Luo, Liancheng Yin, Wenchao Bai, Keming Mao

**Affiliations:** College of Software, Northeastern University, Shenyang 110004, China; 1971141@stu.neu.edu.cn (Y.L.); 1971179@stu.neu.edu.cn (L.Y.); 20185085@stu.neu.edu.cn (W.B.)

**Keywords:** incremental learning, lifelong learning, catastrophic forgetting

## Abstract

As a special case of machine learning, incremental learning can acquire useful knowledge from incoming data continuously while it does not need to access the original data. It is expected to have the ability of memorization and it is regarded as one of the ultimate goals of artificial intelligence technology. However, incremental learning remains a long term challenge. Modern deep neural network models achieve outstanding performance on stationary data distributions with batch training. This restriction leads to catastrophic forgetting for incremental learning scenarios since the distribution of incoming data is unknown and has a highly different probability from the old data. Therefore, a model must be both plastic to acquire new knowledge and stable to consolidate existing knowledge. This review aims to draw a systematic review of the state of the art of incremental learning methods. Published reports are selected from Web of Science, IEEEXplore, and DBLP databases up to May 2020. Each paper is reviewed according to the types: architectural strategy, regularization strategy and rehearsal and pseudo-rehearsal strategy. We compare and discuss different methods. Moreover, the development trend and research focus are given. It is concluded that incremental learning is still a hot research area and will be for a long period. More attention should be paid to the exploration of both biological systems and computational models.

## 1. Introduction

Incremental learning (IL) refers to a learning system that can continuously learn new knowledge from new samples and can maintain most of the previously learned knowledge. IL is a special scenario of machine learning technology, which can deal with applications that are more consistent with human behavior and thinking. Since the new knowledge and the already learned knowledge do not always satisfy i.i.d, this puts forward higher requirements for incremental learning. The classical machine learning model is learned with static, identically distributed and well labeled training data [1]. However, the external environment of the real world is dynamically changing, which needs the intelligent agent to have the ability of continuous learning and memorizing. An incremental learning model can learn new knowledge and retain the old one in lifelong time. It works like a brain system of an organism and it is one of the ultimate goals of artificial intelligence systems. In recent years, it has played increasingly important roles in fields of intelligent robots, auto-driving and unmanned aerial vehicles, etc. [2,3,4].

When learning with new knowledge, the parameter weights are adjusted by a backpropagation algorithm according to loss on available sequential data. This will significantly lower the model performance on knowledge that was learned previously. This is known as catastrophic forgetting (CF), which is the long-standing challenge in incremental learning. To overcome CF, a model must be both plastic to acquire new knowledge and stable to consolidate existing knowledge, but meeting these two requirements at the same time is very hard. This phenomenon is called the stability-plasticity dilemma [5]. The model requires sufficient plasticity to acquire new tasks, but large weight changes will cause forgetting by disrupting previously learned representations. Keeping the network’s weights stable prevents previously learned tasks from being forgotten, but too much stability prevents the model from learning new tasks [6].

In addition to backpropagation, there is another method based on non-iterative learning for model construction. It is highly efficient since it is a globally ordered and locally random learning mechanism [7]. However, for more than 100 related reports reviewed in our work, a non-iterative learning method has not been taken into consideration. Therefore, this article will not focus on the impact of non-iterative training on incremental learning.

Humans show a superior (outstanding) capacity of learning in continuous environments. With long neurophysiological evolutionary progress, the brain has the ability to incrementally acquire and store knowledge over successively sequential tasks. Therefore, the knowledge processing principles of the brain studied through biological ways inspire the development of computational approaches. There exist mechanisms that use to regulate the balance between the stability and plasticity of brain areas and cognitive systems are developed according to external stimulation [8,9]. Hebbian Plasticity was used to describe how neurons respond to external stimuli [10]. It is assumed that when one neuron drives the activity of another, the connection between them will be strengthened. Hebbian plasticity can be enhanced with synaptic constraints and feedback signals [11,12]. The theory of complementary learning system was proposed in [13]. It shows that the hippocampus learns rapidly with short term adaptability, and the cerebral cortex could learn slowly with long term memory. Based on the above hypothesis, many works have been proposed. The influence of catastrophic forgetting for DNNs was studied by Goodfellow et al. and the dropout method was recommended [14]. Kirkpatrick et al. evaluated the importance of parameter weight and proposed that the model stability can be ensured based on weight regularization [15]. Distillation was used to integrate new and old knowledge [16]. Old data was retained and playback was added when necessary [17]. Some researchers conducted surveys on incremental learning methods. In [18,19], methods are introduced based on different types of structures and they mainly focus on model description. However, the existing researches are somewhat dated and lack in-depth (thorough) analysis especially for overcoming CF.

In order to fill this gap, this paper gives an appraisal of incremental learning methods based on the latest reports. Taking CF as the core problem, a systematic description and analysis are given. Works for review are first collected from well known academic engines, including DBLP, Web of Science, etc. (in Section 2). Different scenarios are described for better understanding the problem that incremental learning solved (in Section 3). Each work is reviewed. Then categorization and metrics are given (in Section 4). Benchmark datasets that are commonly used are listed in detail (in Section 5). Moreover, comparisons are given based on their property and performance (in Section 6). Finally, future development trends of incremental learning methods and techniques are shown with our careful consideration.

## 2. Material Selection Criteria

Articles and reports about incremental learning methods from DBLP and the Web of Science were retrieved up to May 2020. This systematic review was done based on the following procedures: (i) relevant keywords are input into specific databases, and retrieval is performed; (ii) repeated works are removed; (iii) each work is grouped based on a set of defined indicators, such as scenario, principle and method type.

The keywords, incremental learning, lifelong learning and continuous learning were used as input for search engines of Web of Science, IEEEXplore, and DBLP. In the initial survey, 353 works were obtained. After identification and removal of the repeated ones, 209 works were selected for the next step. After analysis and selection, 109 articles were identified as the target in this review.

## 3. Incremental Learning Scenarios

**Figure 1:**
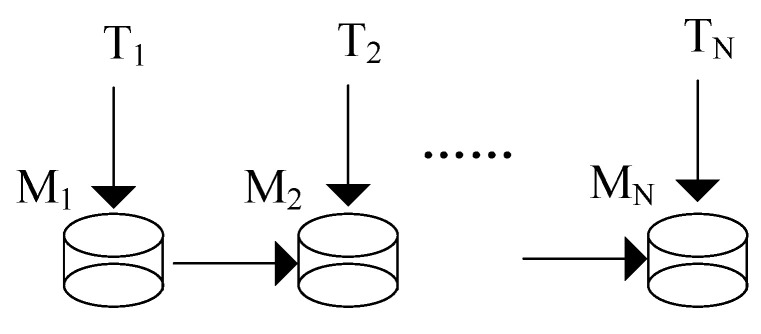
Process of incremental learning.

Incremental learning scenarios are used to describe the context and environment of incremental learning, and it can help us understand the problem and challenges better. van de Ven et al. [20] have provided a comprehensive framework for the scenarios of incremental learning; they classified incremental learning scenarios into task incremental scenario, domain incremental scenario and class-incremental scenario according to specific experimental protocols. This framework is also adopted in [21,22]. However, it does not make a good conceptual distinction between incremental learning scenarios. In fact, the word “task” appears frequently in many studies and then almost all scenarios can be referred to as an incremental task scenario. Figure 1 gives the brief process of incremental learning. The continuously obtained data are divided into sequential tasks, which can be represented as $\left\{T^{1},T^{2},\ldots ,T^{N}\right\}$. $T^{i}$ means *i*-th data group, and $T^{i}=\left\{\left(x_{1}^{i},y_{1}^{i}\right),\left(x_{2}^{i},y_{2}^{i}\right),\ldots \right\}$. The incremental learning model $M_{i}$ is trained with new coming data $T^{i}$ and model $M_{i-1}$, as shown in Equation (1).

(1) $$M_{i}=f\left(T^{i},M_{i-1}\right)$$

In order to avoid concept confusion, we follow [23] to divide incremental learning into three scenarios: instance incremental scenario, class-incremental scenario, instance and class-incremental scenario. In instance incremental scenario, the number of categories is fixed while the data in each category are expanded in each learning stage. In the class-incremental scenario, both the number of categories and incoming data are changed. Of course, the last scenario is the situation where both instances and categories will increase, which is also the most common scenario in the real environment.

Table 1 shows some cutting-edge research directions in the field of machine learning so that we can more intuitively feel the difference between incremental learning and them. Compared with transfer learning, incremental learning requires the model to retain its performance on the old task after learning a new task. In contrast, transfer learning only uses the old knowledge to learn new knowledge. After the learning is completed, it only focuses on the performance of the new knowledge, and no longer considers the performance of the old knowledge.

## 4. Method Description

**Figure 2:**
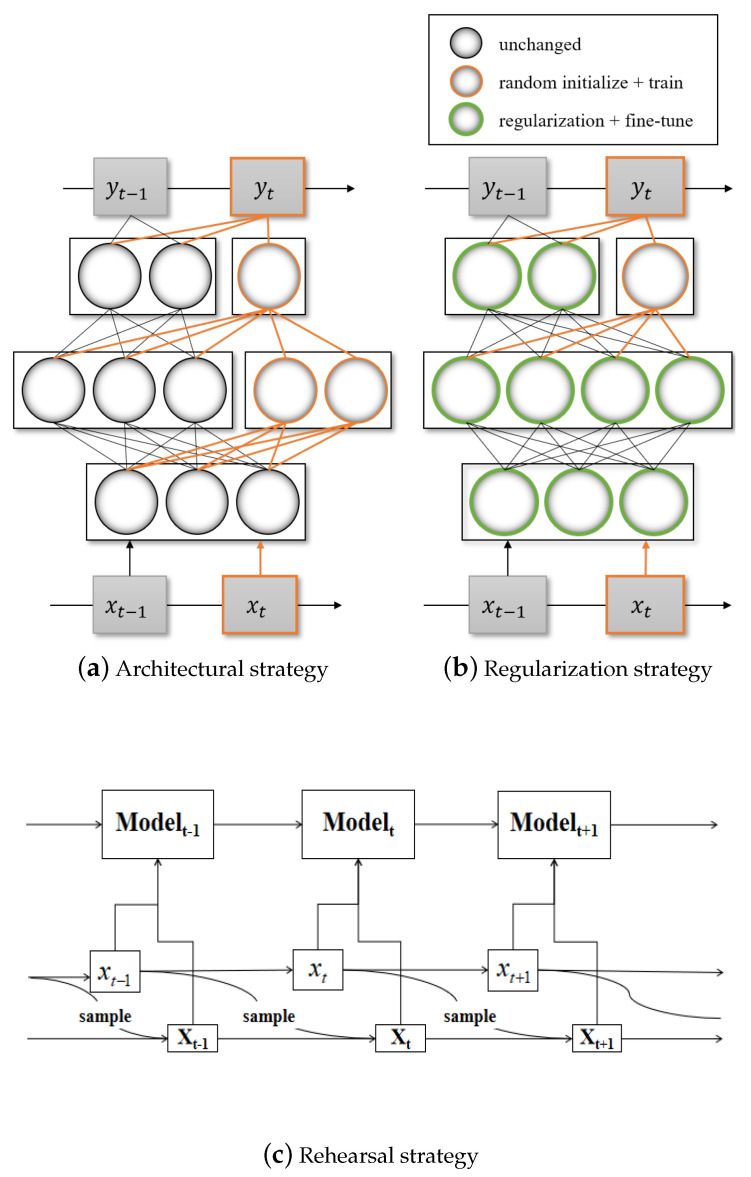

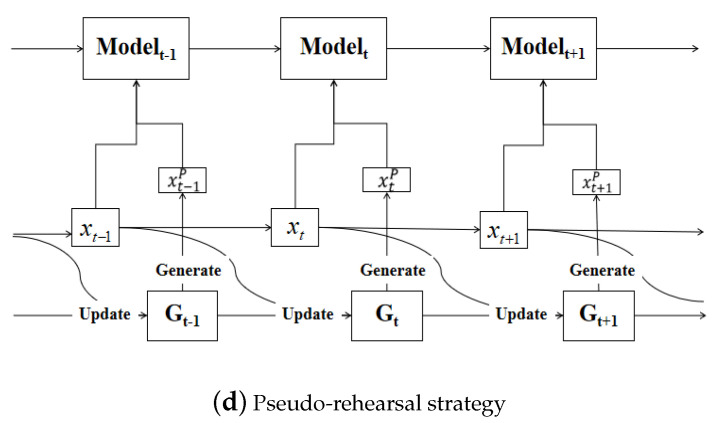
Structure of the following strategies, where *x*${}_{t}$ and *y*${}_{t}$ represent the input and the output at time t, respectively. In (**a**), the network expands with the arrival of new data. In (**b**), the model maintains its original performance through regularization. In (**c**), **X**${}_{t}$ represents the subset at time t, which contains a part of the previous data. In (**d**), **G**${}_{t}$ represents the generator at time t and *x*${}_{t}^{P}$ is a subset generated by it.

Catastrophic forgetting is the key challenge for incremental learning methods. In this research, selected works are reviewed and classified according to different perspectives to solving the problem of catastrophic forgetting in classification tasks. Therefore, three types of solution strategies: architectural strategy, regularization strategy, rehearsal and pseudo-rehearsal strategy are adopted (Figure 2 gives the brief structure of these strategies). It is worth emphasizing that these three strategies do not contradict with each other, instead, they cooperate with each other. What is more, there are also some algorithms designed based on other strategies (in Section 4.4). Finally, comprehensive evaluation metrics will be proposed (in Section 4.5).

### 4.1. Architectural Strategy

In this strategy, separate models are trained for each sequential incremental task. Then a selector is set to determine which model will be used during the inference phase.

Learn++ was proposed by Polikar et al. [24]. It first trains multiple classifiers with different training subsets. Then it makes decisions using a weak classifier combination based on an adaptive boosting neural network. SVM (support vector machine) and learn++ were combined for incremental learning classifier integration [25,26]. As an algorithm proposed earlier, the ability to learn new classes is one of the main features and advantages of learn++. It also has the advantages of a small number of parameters and short training time. However, it suffers from data imbalance when learning new classes.

**Figure 3:**
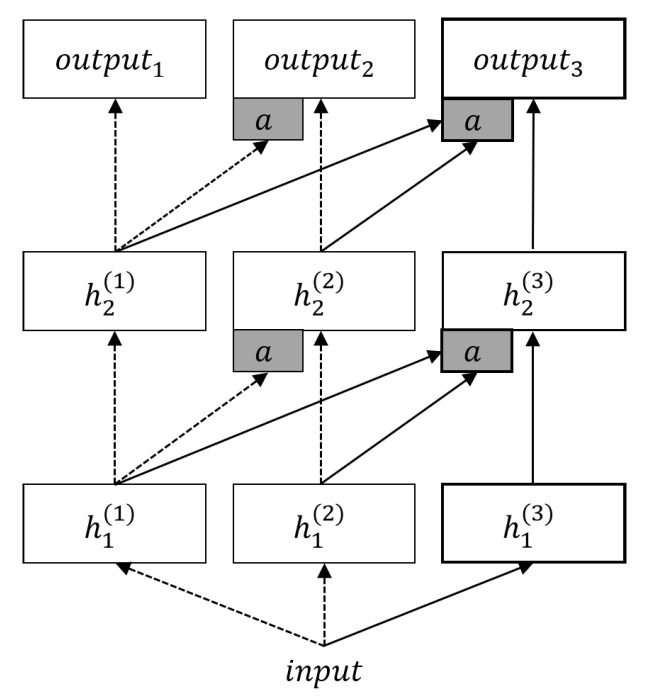
The structure of a progressive neural network (PNN). The two columns on the left (dashed arrow) have been used for training task 1 and task 2 respectively. The gray box marked *a* represents lateral connections to receive the output of the previous network. The rightmost column is added for the last task, which has access to all the features learned before.

A progressive neural network (PNN) model was proposed by Rusu et al. [27]. Parameters of a network trained by previous tasks are fixed to prevent catastrophic forgetting. When training a new task, PNN introduces the experience of previously learned knowledge by taking the output of the previous network into consideration. The structure of PNN is shown in Figure 3. PNN retains the structure that has been trained to protect the performance of the model on the old task and effectively alleviate catastrophic forgetting. However, the number of parameters will gradually increase as tasks increase, and the design of different tasks requires manual intervention.

A hierarchical network with a tree structure was designed by Roy et al. [28]. Incremental learning can be realized by adjusting the leaves of the tree adaptively. Although this method mitigates catastrophic forgetting to a certain extent, it is intuitively a method that consumes more space and is not efficient in training.

Since the methods of expanding the network like PNN cannot make good use of the network capacity, how to use the network more effectively and save space consumption has been further studied.

Overlapping knowledge between stages was learned, and the network structure can be dynamically determined [29]. This dynamic expansion network (DEN) based on correlation can make more efficient use of network capacity and save storage space.

The ExpertGate model was proposed by Aljundi et al. [30]. The expert network was used in a new task, which was determined by an auto-encoder gate, thus an old task that has the highest similarity to the new task will be selected for training.

The method proposed by Gepperth and Karaoguz [31] used a self-organizing map (SOM) to reorganize a two-dimensional coordinate grid. It was only updated when the input was different from previous tasks, and this way can prevent the model changing quickly. In terms of network expansion, ExpertGate and SOM have similar advantages as DEN.

The Self-Net model was proposed by Mandivarapu et al. [32]. An auto-encoder was used to represent a set of low-dimensional weights, which were learned by different tasks. The pretrained weights were initialized with these low-dimensional representations. Since parameters grow only logarithmically with the number of tasks, Self-Net has achieved good results in storage compression.

Model scalability and sustainability were studied in the IADM method [33]. It embeds the attention parameters of Fisher regularization to gradually match the training neural network in each stage. With the adaptive Fisher regularization, IADM is knowledgeable about the past and present data distribution, which can accurately reflect whether the algorithm utilizes the model capacity efficiently.

An incremental-like random forest was used by Hu et al. [34]. Then a splitting strategy was determined for how to insert internal nodes based on the separation axis theorem. Sarwar et al. [35] designed a deep convolutional neural network model that incrementally grows up with new tasks and the basic backbone was retained and shared for previous tasks. Peng et al. [36] alleviated catastrophic forgetting through nerve pruning and synapse consolidation.

We list some other references that proposed similar methods based on architectural design [37,38,39,40,41,42,43,44], so we will not go into the detail of each.

### 4.2. Regularization Strategy

This strategy mitigates catastrophic forgetting by adding a special regularization term to loss function. The core idea is to limit the updating of parameters to improve the model stability, and thereby alleviate catastrophic forgetting. According to different concerns, regularization strategies can be further divided into two types: weight regularization strategy and distillation strategy.

#### 4.2.1. Weight Regularization Strategy

Weight regularization is a commonly used method to mitigate catastrophic forgetting. Through measuring the importance of weights, the old knowledge can be protected by limiting the learning rate. The loss function is:

(2) $$L\left(\theta \right)=L_{n}\left(\theta \right)+\lambda R\left(\theta_{i}\right)$$

where $L_{n}$ is the loss function of new data, $\lambda_{}$ is a hyperparameter, *R* is the regularization term and $\theta_{i}$ is the important parameters to the old knowledge.

Weights of a neural network model will be updated by back propagation (BP) and stochastic gradient descent (SGD). While in an incremental learning scenario, weights of an old model, which is trained by previous data, are updated to a new version, which is more fit to the new knowledge. This will lead to catastrophic forgetting. By identifying those parameters that have a greater impact on the old task and suppressing their update, the model can protect the old knowledge when learning new knowledge. Therefore, the key of parameter regularization is how to measure the importance of parameters and protect them.

One representative method, elastic weight consolidation (EWC), was used to evaluate the importance of weights through the Fisher information matrix [15]. Information carried by the observable random variable is measured based on the Fisher information matrix. EWC supposes that information of previous tasks should be absorbed by a posterior probability, which reflects the importance of weights. Laplace approximation is used to approximate the posterior as a Gaussian distribution, where the mean is given by the weights learned through a previous task and the variance is given by the diagonal of the Fisher information matrix. By this approximation, let the previous task be *A* and the current task *B*, then the loss function of minimizing EWC is:

(3) $$L\left(\theta \right)=L_{B}\left(\theta \right)+\sum_{i}\frac{\lambda }{2}F_{i}{\left(\theta_{i}-\theta_{A,i}^{*}\right)}^{2}$$

where $L_{B}$ is the loss of *B*, *F* is the Fisher information matrix, λ sets how important the old task is compared to the new one and *i* labels each parameter.

Similarly, the method proposed by Amer et al. [45] combined dynamic information balancing and an EWC for model regularization. EWC can effectively save storage space since it is a way to alleviate catastrophic forgetting without expanding the network and retaining old data. This is also the advantage of all regularization strategies. However, EWC only considers the Fisher information matrix for the final stage, not all the previous stages. Therefore, there will still be the phenomenon of interval forgetting.

The SI method was proposed by Zenke et al. [46]. The importance of weight was judged by calculating the cumulative change of distance difference in a Euclidean space after training new tasks. The bigger value means the weight has greater impact on this task. The per-parameter regularization strength is measured by:

(4) $$\Omega_{k}^{\mu }=\sum_{\nu <\mu }\frac{\omega_{k}^{\nu }}{{\left(\Delta_{k}^{\nu }\right)}^{2}+\zeta }$$

where μ and ν mean task ID, *k* represents the *k*-th parameter, $\omega_{k}^{\nu }$ is the parameter specific contribution to changes in the total loss, $\Delta_{k}^{\mu }$ is to ensure that the regular term has the same unit scale as the loss function, and an additional damping parameter ζ is set to bound the expression in cases where $\Delta_{k}^{\mu }\overset{}{⟶}0$. Compared with EWC, SI measures the importance of parameters more intuitively.

Forgetting and intransigence were considered in the work of Chaudhry et al. [47]. The RWalk method was proposed by combining EWC and SI. The Fisher information matrix was calculated based on the last update with the moving average method, and this way can improve the efficiency. Moreover, RWalk adopted approximation KL divergence between output distributions as the distance to calculate sensitivity [48]. In addition to a regularization term, a subset of previous data was also retained. Rwalk improves the regularization methods of EWC and SI, and further improves the performance based on them. However, for performance reasons, some old data are still retained. This shows the effectiveness of rehearsal strategies for mitigating catastrophic forgetting.

The MAS method was proposed by Aljundi et al. [49], which was partly similar with SI but it supports the use of unlabeled data to obtain weight sensitivity. Through unlabeled data, it measured the importance of weight based on model sensitivity, which was obtained by comparing outputs of original and disturbed training data. The importance weight $\Omega_{ij}$ for parameter $\theta_{ij}$ can be shown as:

(5) $$\Omega_{ij}=\frac{1}{N}\sum_{k=1}^{N}\left|\right|g_{ij}\left(x_{k}\right)\left|\right|$$

where $g_{ij}\left(x_{k}\right)$ is the gradient of the learned function with respect to the parameter $\theta_{ij}$ evaluated at the data point $x_{k}$, *N* is the total number of data points at a given phase. Furthermore, Aljundi et al. also proposed an incremental learning algorithm based on MAS [50].

The OWM method was proposed by Zeng et al. [51]. It protected previously learned knowledge by constraining the updated direction of the parameter weights. Specifically, when training a model for continuous new tasks, it only updated the weights in a direction orthogonal to the previously trained feature subspace spans.

The OGD method was proposed by Farajtabar et al. [52]. Every time a new task was coming, the OGD first calculated the orthogonal basis *S* of the old task, and then changed the original gradient of the new task to a new gradient orthogonal to *S*. OWN and OGD update gradients based on orthogonal constraints, which is an intuitive and effective way to maintain model stability. However, they still cannot avoid the limitations of the regularization strategy itself.

Choi et al. [53] proposed an incremental learning method based on a self-encoder, using SI and MAS regularization strategies to alleviate catastrophic forgetting, respectively. They extracted the prototype of the output values of a convolutional layer by an autoencoder and adopted the nearest neighbor classification. Since it only stores the mean prototypes per class, it consumes less storage space than the rehearsal strategy.

The incremental moment matching (IMM) method was proposed by Lee et al. [54]. They used the Bayesian neural network framework to introduce the uncertainty of parameters and calculate the posterior distribution. The dimension of the random variable in the posterior distribution is the number of parameters in the neural network. It approximates the Gaussian posterior mixture, where each component represents the Gaussian distribution of the parameters from a single task to a combined task. Moreover, to make the assumption of Gaussian distribution for neural network reasonable, they applied three main transfer learning techniques on the IMM procedure, which is a major feature of this paper.

A visualization method was used to analyze the catastrophic forgetting in incremental learning [55]. It first divided the neural network into multiple modules. Then it paid attention to which layers are forgotten. Finally, a module can be found that was more plastic and it was frozen while learning the new task to alleviate catastrophic forgetting.

Coop et al. [21] proposed a method that introduced a fixed expansion layer and a hidden layer with sparse coding to overcome catastrophic forgetting. Adversarial losses were used in Singh’s work [56]. Both the architectural strategy and regularization strategy were combined in Maltoni et al.’s work [57]. A task-based hard attention mechanism was designed in Serra et al.’s work [58]. Fisher information was approximated with a diagonalized parameter, and EWC was adopted to mitigate CF [59].

We list some other references that proposed incremental learning methods based on the weight regularization strategy [60,61,62,63,64,65], so we will not go into the detail of each.

#### 4.2.2. Distillation Strategy

Distillation is a macro-protection oriented regularization method, which constrains the output value of the old model and new model. This can make the new model consistent with the old model when learning new data, and knowledge contained in an old model can be drawn into the new model and CF can be partly overcome. Knowledge distillation (KD), proposed by Hinton et al. [66], was originally used to reduce the loss when transferring knowledge from a complex model to a simple model. Through the softmax output layer, the equation of KD can be expressed as:

(6) $$q_{i}=\frac{exp(z_{i}/T)}{\sum_{j}exp\left(z_{j}/T\right)}$$

where *q_i_* is the probability of *i*-*th* class, *z* is the logit of the previous layer. *T* is a temperature coefficient that is normally set to 1. Using a higher value for *T* produces a softer probability distribution over classes.

It becomes one of the most commonly used techniques for incremental learning.

The LwF method was proposed by Li et al. [16]. It trained a separate classifier for each incoming task. The data of the new task was labeled based on the output obtained by the old model (classifier), and these labels were used to constraint the update of the model parameter for knowledge distillation. LwF was the earliest method to apply knowledge distillation to incremental learning. Algorithm 1 gives the design details of LwF. Since then, knowledge distillation has been increasingly applied in various incremental learning methods.
**Algorithm 1** Learning without forgetting**Start with:**
  $\theta_{s}$: shared parameters
  $\theta_{o}$: task specific parameters for each old task
  $X_{n}$, $Y_{n}$: training data and ground truth on the new task
**Initialize:**
  $Y_{o}\leftarrow CNN\left(X_{n},\theta_{s},\theta_{o}\right)$       // compute output of old tasks for new data   $\theta_{n}\leftarrow RANDINIT(|\theta_{n}\left|\right)$    // randomly initialize new parameters
**Train:**
  Define ${\hat{Y}}_{o}\equiv CNN\left(X_{n},\theta_{s},\theta_{o}\right)$       // old task output
  Define ${\hat{Y}}_{n}\equiv CNN\left(X_{n},\theta_{s},\theta_{n}\right)$       // new task output
  $\theta_{s}*,\theta_{o}*,\theta_{n}*\equiv \underset{\theta_{s}*,\theta_{o}*,\theta_{n}*}{argmin}(\lambda_{o}L_{old}\left(Y_{o},\hat{Y_{o}}\right)+L_{new}\left(Y_{n},\hat{Y_{n}}\right)+R\left({\hat{\theta }}_{s}+{\hat{\theta }}_{o}+{\hat{\theta }}_{n}\right)$


Based on LwF, Hao et al. [67] focused on solving object detection problems in incremental learning. A simple encoder model EBLL was designed to characterize each task [68]. The P&C model was proposed in Schwarz et al.’s work [69], which combined the EWC and KD methods.

Hou et al. [70] proposed the DR method. It first trained a separate model for a new task, and then distilled the knowledge of the new model and the old model into a student model by means of knowledge distillation. Since the model is separately trained for a new task each time, various tasks can be effectively learned. In addition, this method shows that keep a small subset of old data has a significant effect on mitigating the CF.

The AFA method was proposed by Yao et al. [71]. It disassembled a complex problem into several simple ones. Two additional loss items using soft labels are added to the loss function, which are low level visual feature alignment and high-level semantic feature alignment. For low level feature alignment, adversarial attention feature maps generated by mapping the same data through non-updated and updated models were used. For high-level semantic feature alignment, Maximum Mean Discrepancy was used [72]. Compared with LwF, AFA improves the distillation loss and increases the restraint of the model, but it does not significantly improve the performance of the model.

The MEDIC method was proposed by Kim et al. [73], which used a maximum entropy regularizer for distillation loss [74], and excluded a number of samples in the new group of classes during stochastic gradient descent of a mini-batch for reducing data imbalance. Compared with other methods, MEDIC has conducted a more comprehensive and detailed evaluation of the performance of the model, including: average task accuracy, forgetting measure and intransigence.

The KT method was proposed by Jung et al. [75], which did not require knowing whether the input data come from the old task or the new task in advance. When training the new data, KT freezes the softmax classification layer, and uses the L2 norm to regularize the feature map between the old and new model. However, this requires an equal number of categories in the old and new tasks.

A global distillation, GD, was proposed in [76]. It first trained a model for a new task, and consolidated the old model and current model by knowledge distillation. A small subset of old data was retained. In addition, an external dataset was build using confidence sampling and random sampling. Finally, a parameter of the classification layer was fine-tuned to avoid over-fitting on the current task. Compared with LwF, GD strengthens the binding force of distillation loss, which helps to improve model stability, but also loses some efficiency. Using unlabeled external datasets has been a promising method in recent years, which was also adopted in [77].

Xiang et al. [78] proposed an algorithm based on dynamic correction vectors to solve the deviation from knowledge distillation and model overfitting problems. Zhao et al. [79] combined weight adjustment and knowledge distillation in order to balance the new and old knowledge. Javed et al. [80] proposed a dynamic threshold shift method to improve the limitations of the deviation in a general knowledge distillation model. Hou et al. [81] integrated cosine normalization, less-forget constraint and inter-class separation into a distillation model to mitigate the negative influences of the imbalance between new and old data.

Other distillation strategy-based incremental learning algorithms can also be referred to in [82,83,84,85].

### 4.3. Rehearsal and Pseudo-Rehearsal Strategy

Rehearsal and pseudo-rehearsal strategies follow a relatively simple idea, retrospection, to deal with catastrophic forgetting. Before the era of deep learning, Robins [86,87] stated that catastrophic forgetting could be mitigated through rehearsal or pseudo-rehearsal. One of the reasons for CF is that incremental learning lacks corresponding supervision for previous knowledge. If a model can review past knowledge when learning new knowledge, it can mitigate catastrophic forgetting. Recently, a study by Knoblauch et al. pointed out that IL algorithms can be seen as polynomial time heuristics targeted at solving an NP-HARD problem and theoretically revealed why a rehearsal and pseudo-rehearsal strategy can more effectively alleviate catastrophic forgetting [88].

Based on this, the rehearsal method allows the model to review the old knowledge whenever it learns new knowledge by retaining a subset of the previous data. The pseudo-rehearsal method constructs a generator to learn the distribution of input data. In order to deal with the plasticity-stability dilemma, when the model learns new knowledge, the generator will produce a batch of pseudo data that is very close to the old data in distribution. In the retraining stage, the model will be supervised by both pseudo data and new data.

iCaRL was proposed in [17], which combined knowledge distillation and prototype rehearsal technologies. It was designed for a class-incremental scenario, and *m* samples were retained for each class type. Samples were selected based on the closest distance to the prototypes. Moreover, iCaRL set a constant value to total storage of a model prototype. Algorithm 2 gives the specific procedure for removing exemplars. Similarly, EEIL adopted a similar method for retaining old data [89]. Although iCaRL limits memory consumption to some extent, it does not meet the requirements of long-term increments.
**Algorithm 2** iCaRL reduce exemplar set**input** m          // target number of exemplars
**input**
$P=(P_{1},\ldots ,p_{\left|P\right|})$    // current exemplar set   $P\leftarrow (p_{1},\ldots ,p_{m})$       // i.e. keep only first m
**output** exemplar set *P*  


Wu et al. [90] proposed a method of retaining a small subset of old data and knowledge distillation. In addition, in order to make up for the distribution difference between retained exemplars implicit data, they used vanilla generative adversarial networks to learn the distribution of old data, which is easier to implement than conditional GANs when the number of categories is large and the samples are limited [91]. Based on GANs while retaining part of the old data, which enhances its stability. However, more training data usually means longer training time. In addition, the introduction of GANs brings the problem of incremental GANs.

**Figure 4:**
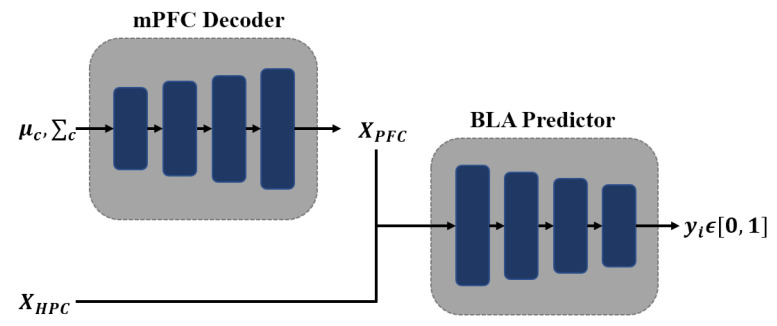
The basolateral amygdala (BLA) sub-systems in FearNet, where $\mu_{c},\Sigma_{c}$ are the base-knowledge of long-term memories and $X_{HPC}$ are the recent memories. BLA is used during prediction time to determine which memory should be recalled from short- or long-term memory.

Inspired by the two-layer memory model mammalian [13], FearNet was proposed in [92]. It includes three networks, probabilistic neural network hippocampal complex (HC) for storing recent memories, autoencoder medial prefrontal cortex (mPFC) for storing long-term memories and basolateral amygdala (BLA) for deciding which networks were selected for recall. Figure 4 gives the BLA sub-systems. Moreover, FearNet contained a sleep phase and prediction phase. The sleep phase was used to train mPFC for memory integration with samples from Gaussian mixture distribution based on the mean and variance of each category. The new sample and the pseudo sample were combined to fine-tune the mPFC. In the prediction phase, the outputs of HC or mPFC were decided for prediction. FearNet has good memory efficiency, and its design of the long- and short-term memory mechanism is more in line with the mammalian brain structure at the biological level. However, it does not involve incremental training of feature extractors, which is also an important issue that needs to be considered for incremental learning.

Shin et al. [93] trained a separate generative model for data rehearsal, which followed variational autoencoder (VAE) [94]. It used KL divergence and autoencoder to approximate the data distribution. Compared with GANs, VAE introduced hidden variables, and it was relatively easy to learn for its linearly theoretical derivation. However, the generated images were more fuzzy.

The BIC method was proposed in [95]. It first pointed out that the last fully connected layer of the neural network has a relatively large deviation for the parameters that were not shared across classes. Then a correction layer was added to rectify the deviation, which is simple and effective in dealing with the data imbalance issue.

DGM was proposed in [96], which relies on conditional generative adversarial networks. It trained a sparse binary mask for each layer of the generator. The learned mask can obscure the model connection plasticity, and it was possible to prevent the important units from being overwritten by restricting the update of parameters. At the same time, DGM also considered the problem of dynamic network expansion. The number of units used in each layer of the generator was appended to ensure the model had sufficient capacity when it trained.

The SIGANN proposed by [97] consisted of three modules, the classifier module, the generator module and the detector module. The joint action of the detector was composed of Meta-recognition and OpenMax, and it can judge whether the input contained new categories. In this way, SIGANN could automatically learn new knowledge when needed. The classifier module included an encoder unit shared with the generator module. The generator module was designed based on an adversarial self-encoder.

Guo et al. [98] proposed an example set exemplar-based subspace clustering method. Riemer et al. [99] used an autoencoder based model to support scalable data storage and retrieval for scalable old data.

Li et al. [22] proposed to balance the generated samples and the new coming data samples by adjusting the training batch. Kim et al. [100] proposed an incremental learning algorithm based on attribute sharing. Shah et al. [101] proposed to eliminate model deviation by distilling knowledge from an Auxiliary classifier.

Other incremental learning methods based on rehearsal and pseudo-rehearsal strategy included [102,103,104,105,106,107,108,109,110,111,112,113,114].

### 4.4. Other Strategies

Besides the three most commonly used strategies mentioned above, there are also some other methods to achieve incremental learning, such as meta learning-related methods and reinforcement-learning-inspired methods. Related works will be covered below.

Wang et al. [115] explored incremental reinforcement learning and proposed a two-step solution incorporated with the incremental learning procedure: policy relaxation and importance weighting. In the initial learning episodes, policy relaxation can encourage the model to explore appropriately in the new environment. During parameter updating, learning episodes receiving higher returns will be assigned higher importance weights for encouraging the previous optimal policy to be faster adapted to a new one that fits in the new environment. This method can help the model adapt to the changing environment faster.

Perez-Rua et al. [116] proposed OpeN-ended Centre nET (ONCE) for solving the problem of incremental object detection and segmentation. ONCE is based on the structure of CentreNet [117] and splits it into a feature extractor and an object locator. It uses meta-learning to train the code generator, outputs the corresponding weight for each category of images, and uses the weight to complete the detection of the test target. Compared with other few-shot detection algorithms, the advantage of ONCE is that after training on the basic dataset, the new small sample dataset can be directly used for inference, and the contents of the basic dataset will not be forgotten in this process. iTAML [118] is also an incremental learning algorithm designed based on meta-learning, but it focuses on solving classification tasks.

Time series anomaly detection is also a common problem faced by incremental learning. Related research was carried out in [119], and they used incremental tensor decomposition to solve the task of online time series anomaly detection.

### 4.5. Evaluation Metric

Although many studies only focus on the improvement of overall accuracy, the evaluation metric of incremental learning should also include efficiency. As argued in [120], focusing only on the problem of forgetting may lead to bias in the research of incremental learning.

Lopez-Paz and Ranzato [102] pointed out that the ability of learners to transfer knowledge should also be paid attention to, and accordingly proposed the concepts of backward transfer (BWT, which is the influence that learning a task has on the performance on previous tasks) and forward transfer (FWT, which is the influence that learning a task has on the performance on future tasks). Given the train-test accuracy matrix $R\in R^{N\times N}$, which contains in each entry $R_{i,j}$ the test classification accuracy of the model on task $t_{j}$ after observing the last sample from task $t_{i}$. For BWT, positive backward transfer can increase the performance on some preceding tasks, and large negative backward transfer is known as CF. Considering the average of the backward transfer after each task, the metric of BWT can be shown as:

(7) $$BWT=\frac{\sum_{i=2}^{N}\sum_{j=1}^{i-1}\left(R_{i,j}-R_{j,j}\right)}{\frac{N(N-1)}{2}}$$

For *FWT*, positive forward transfer is possible when the model is able to perform “zero-shot” learning. The metric of FWT can be defined as:

(8) $$FWT=\frac{\sum_{i<j}^{N}R_{i,j}}{\frac{N(N-1)}{2}}$$

Forward transfer is a challenge worth paying attention to in incremental learning, and research in this area needs to be further deepened.

Take into account the scalability of an incremental learning system, comprehensive evaluation metrics for incremental learning could include [5,20,120,121,122]: accuracy, train/test time, storage size (including model size and samples storage size), whether the algorithm needs task id or not, BWT and FWT.

## 5. Datasets

Incremental learning methods usually adopt generic datasets for evaluation, which include MNIST, CIFAR-100. ImageNet ILSVRC, SVHN, PASCAL VOC, MIT Scenes, Caltech-UCSD Birds and Oxford Flowers. References of the data set can be seen in Table 2.

MNIST is a handwritten digital dataset [123]. It contains 10 classes and a total of 70,000 images. All images are gray level with size of 32 × 32 pixels. MNIST is adopted in [15,22,33,47,52,54,75,92,96].

CIFAR-100 is a common object dataset [124]. It has 100 classes containing 600 images each, and 100 classes are grouped into 20 superclasses. All images are RGB format with size of 32 × 32 pixels. CIFAR-100 is used in [17,47,53,73,76,77,89,90,95].

The ImageNet dataset is collected from flickr and other search engines. It contains 1000 categories and 1.2 million images for training [125]. Image samples in this dataset are not fixed-size. ImageNet is used in [16,17,51,70,71,76,89,95,96].

SVHN is a street view house numbers Dataset [126]. It contains 600,000 digit images that come from Google Street View, and it is a significantly harder, unsolved and real-world problem. The size of image samples is a 32 × 32 pixels RGB format. The authors of [22,75,96] employ this dataset.

PASCAL VOC is a dataset for object classification, detection and segmentation. It has 20 classes, and11,530 images in total containing 27,450 ROI annotated objects and 6929 segmentations [127]. The sizes of image samples are diverse. PASCAL VOC is applied in [67,77,126].

MIT Scenes is an indoor scene recognition dataset [128]. It contains 67 indoor categories and a total of 15,620 images. The number of image samples varies across categories. There are at least 100 images per category. It is used in [16,49,70,71].

Caltech-UCSD Birds 200 (CUB-200) is a challenging image dataset annotated with 200 bird species [129]. In total it has 11,788 image samples, and it is downloaded from Flickr and filtered manually. The authors of [16,49,53,70,71,92] used the dataset.

Oxford Flowers is a dataset used for flower image fine classification [130]. It contains 102 categories and 8189 image samples. Each category includes 40 to 258 images. The studies [49,70,71,90] selected this dataset.

Besides these commonly used generic datasets, the CORe50 dataset was proposed in [23], which is the benchmark for continual Learning and Object Recognition, Detection and Segmentation. It simulates an incremental learning environment for evaluation. Fifty domestic objects belonging to 10 categories are collected. For each object, multiple continuous frames are recorded with smooth moving and rotation. So the classification task can be performed at the object level (50 classes) or at category level (10 classes). The final dataset consists of 164,866 128*128 RGB-D images. CORe50 supports three continuous learning scenarios, New Instances, New Classes, New Instances and Classes, respectively.

Since the research of incremental learning is very important to robotics, there are also some datasets proposed for robotics. In IROS 2019-Lifelong Robotic Vision Competition, OpenLORIS-Object was proposed to promote lifelong learning research and applications in the field of robot vision, including daily necessities in homes, offices, campuses and shopping malls [121,131]. The dataset clearly quantifies the illumination, occlusion, object size, camera-object distance/angle, and clutter. The version of OpenLORIS-Object for this competition is a collection of 69 instances, including 19 categories of daily-necessity objects under seven scenes. The benchmarks of OpenLORIS-Object include the overall accuracy of all tasks and efficiency (model size, inference time and replay size).

## 6. Discussion and Comparison

In this chapter, we will discuss and analyze the advantages and disadvantages of various strategies, and give some comparisons of algorithms. Then, based on the current work, we will summarize the current research and look into the future development direction. Finally, we will look at the role of incremental learning in robotics.

### 6.1. Strategy Discussion

In this part, we discuss the characteristics and shackles of several mainstream incremental learning strategies.

Due to its characteristics, the architectural strategy has a natural advantage in maintaining the stability of the model. However, it requires the model to continue to expand, which means that the parameters will continue to increase with each task. In addition, the incremental learning algorithm based on the architectural strategy usually requires the task identity to be informed in advance during the inference phase [27,29], which restricts the robustness and flexibility of the strategy and makes it difficult to tackle for a more realistic environment.

In terms of the regularization strategy, the thought of reducing the plasticity of neural networks to improve stability is theoretically close to the long-term memory of biological brains. Weight regularization does not require extra storage space and sometimes has a good effect on improving network stability and mitigating catastrophic forgetting. However, the weight regularization strategy struggles quite a lot when the number of tasks is large. This is because after the model has been trained many times, many parameters in the neural network will be protected due to the constraints of regular terms, so that the parameter update becomes increasingly difficult. Moreover, when the new and old tasks are sensitive to the same parameter, it is difficult to balance the update of the parameters. Furthermore, different tasks may be sensitive to the same parameters, and the processing method at this time is also a big challenge.

Compared with weight regularization, the regularization strategy based on knowledge distillation makes the model more plastic. However, the issue that follows is that the supervision of soft labels obtained using new data is not strong enough, and there will be an imbalance between the old and new classes. Many distillation strategy methods can only effectively mitigate catastrophic forgetting when the number of incremental tasks is small, in which it is still difficult to meet the requirements of long-term increments [16,70,71].

Among several strategies for dealing with CF, the rehearsal strategy has the longest history and works well. It is still regarded as an effective strategy to mitigate CF until today. Since CF can be greatly relieved by only retaining a small amount of old data, rehearsal strategy often appears as an effective auxiliary method in various incremental learning methods. Many incremental learning methods based on the regularization strategy save a small amount of old data to enhance model performance [47,70,76]. Although it has a good effect, it also has its own limitations. The main drawback of the rehearsal strategy is that storing old data requires a lot of memory space, but we cannot have infinite space to store enough data. At the same time, more training data usually means lower training efficiency. These challenges could be alleviated by optimizing data storage methods [99], but it still cannot be completely overcome. If the memory capacity is limited [17], the sample size of a single knowledge category will gradually decrease with the accumulation of tasks, and its impact on the model will gradually decrease. In addition, in many cases, due to considerations such as security and personal privacy, old data is not allowed to be retained.

With the maturity of generative adversarial networks, the pseudo-rehearsal strategy has recently received increasing attention. Comparing with the rehearsal strategy, the pseudo-rehearsal strategy does not need to save a large number of real samples, and has great advantages in protecting privacy and saving memory. However, the pseudo-rehearsal strategy requires the use of a generative model that can meet the incremental requirements, which currently seems more difficult than implementing incremental learning. Unless it is assisted by using data with real samples [132], some methods can hardly present a satisfactory performance [93]. Moreover, it is difficult for the current generator to generate complex pictures, which means the pseudo-rehearsal strategy can only achieve results in some simple classification problems. Seff et al. [133] proposed to use EWC in the generative network to avoid repeated training of the generator each time, which is an exploration of the incremental generator.

As each strategy has corresponding limitations, researchers increasingly tend to combine various strategies to achieve incremental learning.

### 6.2. Algorithm Comparison

In this part, we will describe Table 3. Table 3 is a general comparison of some incremental learning methods mentioned in Section 4. Considering that the experimental results are closely related to the experimental protocol, in order to facilitate comparison, we chose the experimental protocol settings as similar as possible, and selected the results obtained by the relatively more general design in the experimental results section. Nevertheless, since most algorithms only focus on accuracy without a comprehensive evaluation mentioned in Section 4.5, Table 3 only collects results related to accuracy, and a more direct comparison can be found in [20,122].

By observing relevant experimental protocols and comparing experimental results, we can observe that:

- Although the experimental protocol of each reference is not exactly the same, incremental learning is the basic research to realize artificial intelligence, so the selection of the datasets is often close and general. Some general datasets are frequently used, such as MNIST, CIFAR-100, and ImageNet ILSVRC.
- The experimental protocol of some methods is closer to the instance incremental scenario, and all have obtained relatively good experimental results [15,33]. It shows that the implementation of an instance incremental scenario is less difficult than the class-incremental scenario. In addition, EWC, as a representative method of weight regularization, can also be used in a class-incremental scenario, which indicates that the regularization strategy is applicable to both an instance incremental scenario and class-incremental scenario.
- Aljundi et al. [49] compared the classification performance between a multi-head classifier and single-head classifier. It can be seen that a multi-head classifier can achieve higher classification accuracy than a single-head classifier. For some low-complexity data, the incremental learning algorithm using a multi-head classifier can obtain quite good results. However, the multi-head classifier requires the task identity to be informed in advance during the inference stage, which is a strict condition that limits its versatility.
- The experimental results of [47,70,76] prove that the algorithm based on the regularization strategy is appropriately combined with a rehearsal or pseudo-rehearsal strategy, which is of great help to improve the performance of the model. This is because the essence of current artificial intelligence technology may still be data fitting, and data support is the basis of fitting.
- The dual memory system designed by FearNet [92] conforms to the mammalian memory system and is a model design method worth exploring. However, it directly uses pre-trained ResNet embeddings as extracted features that feed to FearNet, which makes the model’s ability to extract features in new data questionable.
- Using similar experimental settings, the gap between the results of the three methods based on the distillation strategy is not too obvious [16,70,71]. This means that the supervision ability of soft labels has not been significantly enhanced, i.e., the method of using only new data is not reliable at present.

At present, the rehearsal strategy is still the optimal solution for dealing with CF, although there are still disputes in the academic circles about whether incremental learning should strictly limit the use of old data. Based on the collated multiple pieces of incremental learning materials and practical application considerations, this paper believes that incremental learning should not be limited to whether it can review old data. For incremental learning, the most fundamental requirement is the ability to learn new knowledge efficiently and autonomously while resisting catastrophic forgetting. Unless required by the actual environment, any restrictions beyond that are unnecessary.

### 6.3. Trend Analysis and Prospects

In this part, we analyze the research trends of incremental learning and explore its prospects based on the current situation.

**Figure 5:**
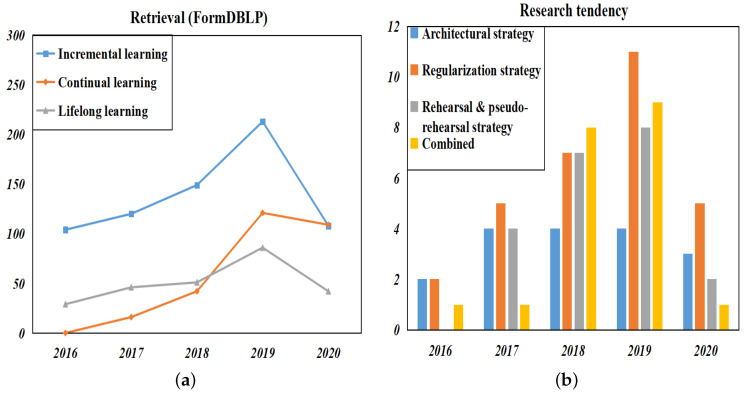
Research status of incremental learning: (**a**) retrieval of incremental learning (lifelong learning, continual learning) in DBLP in the last five years, (**b**) the use of various incremental learning strategies over the past five years.

Through statistics on the publication of incremental learning (continuous learning, lifelong learning) in DBLP and the strategies used by various incremental learning algorithms in Section 4, we have calculated the incremental learning research trend from 2016 to 2020, as shown in Figure 5.

As shown in Figure 5a, with the development of deep learning, the academic circles have regained the research enthusiasm for incremental learning in recent years. The development of databases and Internet technology has made the acquisition and updating of data increasingly rapid. People urgently need a model that can continuously effectually learn useful new content from massive amounts of data without losing the original performance. Moreover, incremental learning is an important part of realizing true intelligence. After all, a system that cannot continue to learn is not a truly intelligent system, and the cost that comes with it is that it always requires manual intervention. Therefore, incremental learning is still an important challenge to be solved in the future.

In terms of various strategies to deal with CF, it can be seen from Figure 5b that the regularization strategy has received widespread attention at present, the research on the architectural strategy tends to be stable, and the research on rehearsal and pseudo-rehearsal strategies is gradually increasing.

Regularization strategies are attractive since they are efficient and require no extra storage. The pseudo-rehearsal strategy has more possibilities due to the development of GANs.

What is more, the proportion of attempts to the combined strategy is gradually increasing, which implies that the current research on incremental learning has fallen into a bottleneck.

For the combination strategy, the combination of the distillation strategy and rehearsal strategy is the most popular. On the one hand, only a small amount of old data needs to be retained to greatly alleviate CF [70,73]. On the other hand, the rehearsal strategy can make up for the lack of soft label supervision in the distillation strategy, and the distillation strategy can improve training efficiency and reduce space consumption.

In addition to being a bottleneck in strategy, the existing incremental learning algorithms generally suffer from poor flexibility and strict environmental requirements. Since the current research on incremental learning is almost task-based, the experimental protocol is strict, which cannot simulate the real environment very well. In the real environment, data flows are coming constantly, and there is no obvious boundary between the data, i.e., the boundaries of the task will not be predefined. Hence, a more realistic incremental learning environment should be task-free. This requires the model to be able to automatically determine when to perform incremental training, and effectively mitigate CF while incorporating new knowledge into the system. Aljundi et al. [50] have explored the design of this system.

The universal applicability of incremental learning makes it occupy an important position in the research of other machine learning fields, such as incremental transfer learning [102], incremental reinforcement learning [115], and so on. In the future, combining various strategies is still the mainstream, and incremental learning systems in a variety of environments will be explored. Moreover, the introduction of decremental learning may help incremental learning systems to achieve long-term increments with existing technology and limited capacity.

Decremental learning means removing unimportant (or long-term unused) knowledge from the system to make space for learning new knowledge.

The working mechanism of the human brain is inspiring for the study of neural networks. For incremental learning, many articles have emphasized that catastrophic forgetting does not occur in the human brain, but have ignored the fact that forgetting can also occur in the human brain. Even if long-term memory is formed by creating new synapses, the human brain will forget it after a long period of inactivity. This inspired us to consider the forgetting phenomenon in the design of incremental learning. For machines, if the forgetting mechanism can be triggered dynamically based on the surrounding environment and received data, then it is theoretically possible for the existing technology to achieve long-term increments without unlimited expansion of the model.

### 6.4. Incremental Learning for Robotics

Finally, in terms of applications, the real-world applications of incremental learning are almost limitless. In fact, any system involving continuous learning requires the participation of incremental learning. The advent of the 5G (5th generation mobile networks) makes the speed of information circulation even further, and in the era of big data, the importance of incremental learning will become more prominent. In the field of big data processing, intelligent robots and any field involving knowledge updates, incremental learning will play an important role. Among them, applications on robotics or autonomous systems are the most intuitive application fields for incremental learning.

A lifelong vision challenge for assistive robotics was firstly introduced by Mozaffari et al. [2]. In practice, assistive robots should be able to operate in dynamic environments with everyday changes. The variations include illumination, occlusion, camera-object distance/angles and clutter. Through testing based on the OpenLORIS-Object dataset, they found that the three most adopted regularization methods in lifelong learning (EWC, SI and LwF) have little effect on solving the lifelong vision challenges for assistive robotics. The research reveals that the current application of incremental learning in assistive robots is still far from enough, and algorithms that can tackle these practical factors urgently need to be developed.

Simultaneous Localization and Mapping (SLAM) is one of the core problems in the field of robotics, which aims to enable the robot to autonomously estimate its own position and posture during the movement. Liu et al. [137] firstly introduced “lifelong SLAM” to distinguish SLAM in static settings from in an ever-changing environment. Lifelong SLAM emphasizes the positioning failure and mismatch problems caused by scene changes, which could be addressed by incremental learning. They released the OpenLORIS-Scene datasets (datasets that emphasize scene change) to accelerate lifelong SLAM research.

Lesort et al. [138] summarized incremental learning in the context of robotics, made a comprehensive review of incremental learning for robotics. There are three important incremental learning use cases on robotics: perception, reinforcement learning (RL) and model-based learning. Perception includes classification, object detection and semantic segmentation, which are all concerns in the current incremental learning research field. In the real world, the constantly changing environment poses more daunting challenges to the perception of agents. Incremental learning is crucial to address these challenges. In the context of reinforcement learning, in order to learn an approximately stable data distribution, techniques similar to those proposed in incremental learning are often used, such as the rehearsal method [139]. Model-based learning is a form of reinforcement learning, and its high data utilization rate makes it popular in robotics applications. In [140], Raffaello et al. presented an approach for incremental semiparametric inverse dynamics learning, which used parametric modeling based on rigid body dynamic equations and nonparametric modeling based on incremental kernel methods.

## 7. Conclusions

This paper makes an appraisal of incremental learning methods. We introduced the basic concepts and main challenges of incremental learning, and analyzed three incremental learning strategies to mitigate catastrophic forgetting: architectural strategy, regularization strategy and rehearsal and pseudo-rehearsal strategy. Through the discussion and comparison of related incremental learning methods, we analyzed the current research situation of incremental learning and looked forward to the future incremental learning research from the aspects of application and theory. Although the current work has made good progress, the realization of flexible and stable incremental learning that can adapt to various complex environments is still far away. Through analysis, we found that the strategy for dealing with catastrophic forgetting has reached a bottleneck. Researchers are increasingly inclined to use a combination of various existing strategies to study incremental learning, which is usually better than using a single strategy alone. The lack of flexibility and practicality are the dilemmas faced by many current incremental learning methods. Most methods are task-based designs, but the actual data flow is much more complicated than this. Therefore, the incremental learning of task-free design should be considered more. One possible way is to introduce unsupervised learning. Since the iterative update of parameters with the arrival of new data is a major internal cause of catastrophic forgetting, non-iterative training may be able to overcome catastrophic forgetting from a lower level, which is also a direction worthy of attention in the future.

This is the first step in our research on incremental learning. After this, we will focus on the interpretability of incremental learning and the underlying design issues, to explore the possibility of achieving incremental learning from the basic level.

## Figures and Tables

**Table 1:** Definitions of different types of learning.

Type	Definition
Incremental learning	Continuously and efficiently learn new knowledge while maintaining the performance of the model on old knowledge.
Transfer learning	Apply the knowledge of a solved problem to a different but related problem.
Meta-learning	Meta-learning aims at mastering the ability to learn so that an agent can master many tasks.
Multi-task learning	Learn multiple related but different tasks at the same time.
Few-shot learning	Dataset contains only a limited number of examples with supervised information for the task.

**Table 2:** References of the dataset.

Datasets	Reference
MNIST [123]	[15,22,33,47,52,54,75,92,96]
CIFAR-100 [124]	[17,47,53,73,76,77,90,95]
ImageNet ILSVRC 2012 [125]	[16,17,51,70,71,76,89,95,96]
SVHN [126]	[22,75,96]
PASCAL VOC 2012 [127]	[16,67,77]
MIT Scenes [128]	[16,49,70,71]
Caltech-UCSD Birds 200 [129]	[16,49,53,70,71,92]
Oxford Flowers [130]	[49,70,71,90]
CORe50 [23]	[122]
OpenLORIS-Object [121]	[131]

**Table 3:** General comparison of some incremental learning for image classifications.

Reference	Strategy	Dataset	Method	Compare the Result with	Result
Kirpatirck et al. [15]	Weight regularization	They permuted MNIST into 10 subsets	EWC uses the Fisher information matrix to evaluate the importance of weights	-	Average accuracy is 94.5%
Zenke et al. [46]	Weight regularization	They divided MNIST into 5 subsets of consecutive numbers	SI evaluates the importance of weights by calculating the cumulative change in European unit loss before and after parameter updating	EWC [15]	A multi-head approach is adopted, and average accuracy is 97.5%
Chaudhry et al. [47]	Combined	They divided MNIST and CIFAR-100 into 5 and 10 disjoint subsets based on classes	Rwalk improves on the basis of EWC and SI	EWC, SI [46], iCaRL [17]	Multi-head classifier: Accuracy (MNIST) = 99.3% Accuracy (CIFAR-100) = 74.2% Single-head classifier: Accuracy (MNIST) = 82.5% Accuracy (CIFAR-100) = 34.0%
Aljundi et al. [49]	Weight regularization	CUB-2011, MIT Scenes, Oxford Flowers: They train a multi-head classifier to classify them in increments of the dataset	MAS evaluates the importance of weights based on the sensitivity of functions that have been learned after parameter changes	LwF [16], EWC, SI, EBL [68]	Accuracy: (Birds→Scenes) 53.20% and 55.0% respectively (Flower→Birds) 76.63% and 50.39% respectively
Yang et al. [33]	Weight regularization	They divided MNIST into 4 subsets	IADM uses an improved Fisher information matrix	EWC, DEN [29]	Average accuracy is 89.2%
Choi et al. [53]	Combined	CIFAR-100, CUB-2011: The initial training subset contains half classes in a dataset, and each other contains a single class	Based on SI and MAS, while retaining some old data after encoding	FearNet [92], iCaRL	In CIFAR-100, average accuracies are 85.0% (MAS) and 85.7% (SI) In CUB-2011, average accuracy are 76.9% (MAS) and 76.2% (SI)
Farajtabar et al. [52]	Weight regularization	They divided MNIST into 5 subsets, each subset contains two classes	OGD limits the direction of weight update	EWC, A-GEM [134]	A multi-head approach is adopted, average accuracy is 98.84%
Rebuffi et al. [17]	Combined	They split CIFAR-100 and train all 100 classes in batches of 5, 10 or 20 classes at a time	iCaRL retains a subset of old data and combines KD techniques	-	The average classification accuracies are 62.1%, 64.5% and 67.5%, respectively
Wu et al. [90]	Combined	CIFAR-100: Similar settings to [17]	Combined iCaRL and LwF, and used GANs to expand the training set	iCaRL	The average classification accuracies are 63.85%, 66.05% and 68.44%, respectively
Kemker et al. [92]	Pseudo-rehearsal	CIFAR-100, CUB-2011: They set the batch size to 10 classes	FeaNet is inspired by the long-term and short-term models of mammalian memory	FEL [21], iCaRL, GeppNet [31]	Average accuracies are 94.7% (CIFAR-100) and 89.1% (CUB-2011)
Li et al. [96]	Combined	MNIST, SVHN, CIFAR-10: They divided these datasets into 10 subsets, each subset contains 2 classe	DGM uses GANs and designs a dynamic network expansion method	EWC-M [133], iCaRL, MeRGAN [135], DGR [93]	The accuracy rates on MNIST, SVHN and CIFAR-10 are 98.75%, 83.93% and 64.94%, respectively
Mellad et al. [97]	Pseudo-rehearsal	They divided EMNIST [136] into 4 subsets, each subset contains 9 classes	SIGANN’s generator is based on adversarial autoencoder and has a detector to determine whether the model needs to be updated	-	The average accuracy is 70.11 ± 2.21%
Deboleena et al. [28]	Architectural	They divided CIFAR-100 into 10 subsets, each subset contains 10 classes	Tree-CNN adopts an adaptive hierarchical network structure	iCaRL, LwF	To Tree-CNN-5 (maximum number of child nodes for a branch node is set at 5), the final test accuracy is 61.57%
Jaehong et al. [29]	Architectural	They divided CIFAR-100 into 10 tasks, and set each task as a set of 10 subtasks	DEN dynamically determines its network structure through a series of stages	EWC, PNN [27]	Average accuracy is 92.25%
Kim et al. [73]	Combined	CIFAR-100, TinyImageNet (a subset of ImageNet 2012): Similar setting to [28]	MEDIC uses maximum entropy as distillation loss, while retaining some old data	EWC, EEIL	Average accuracy is 72.51 ± 0.17%
Wu et al. [95]	Combined	They divided ImageNet(2010) into 10 subsets	BiC adds a correction layer based on iCaRL and EEIL [89]	LwF, iCaRL, EEIL	Average accuracy is 73.2%
Lee et al. [76]	Distillation	Similar to [73]	GD designs a loss called Global Distillation and uses unlabeled external data to assist training	LwF, EEIL	Average accuracies are 68.1 ± 1.1% (CIFAR-100) and 57.7 ± 1.6% (TinyImageNet)
Li et al. [16]	Distillation	ImageNet ILSVRC 2012, CUB-2011, MIT Scenes: The datasets are used in a similar way to [49]	LwF uses KD to mitigate catastrophic forgetting	-	Accuracy: (ImageNet→CUB-2011) 54.7%, 57.7% respectively (ImageNet→MIT Scenes) 55.9%, 64.5% respectively
Hou et al. [70]	Distillation	Similar to [16]	Improved the method of knowledge distillation based on LwF	LwF	Accuracy: (ImageNet→CUB-2011) 55.34%, 58.21% respectively (ImageNet→MIT Scenes) 55.65%, 64.70% respectively
Yao et al. [71]	Distillation	Similar to [16]	AFA adds two regularization terms using soft labels to the loss function based on LwF	LwF, EBLL, MAS [49], EWC	Accuracy: (ImageNet→CUB-2011) 54.43%, 57.84% respectively (ImageNet→MIT Scenes) 54.71%, 63.88% respectively
